# Annual Commencement

**Published:** 1849

**Authors:** 


### ARTICLE IX.

### ANNUAL COMMENCEMENT.

At the annual Commencement of Rush Medical College, held on the 22d of February, 1°49, the degree of Doctor of Medicine was confered on the following named gentlemen, who had complied with the requirements of the Institution, and passed a satisfactory examination:

| *Names.* | *Title of Thesis.* |
| --- | --- |
| Alfred W. Armstrong, | On Erysipelas. |
| Wm. W. Cavarly, | On Intermittent Fever. |
| Asa Clark, | On Purpura Hemorrhagica. |
| Harvey Cutler, | On Narcotics, |
| Joseph W. Freer, | On Anæsthetic Agents. |
| Charles C. Garrett, | On Amenorrhoea connected with Chronic Hepatites. |
| Israel G. Harlan, | On Eutozoa. |
| George M. Huggans, | On Ergot as a Parturient Agent. |
| Calvin B. Lake, | On Continued Fever. |
| Robert Pennell Lamb, | On Typhoid Fever. |
| Orrin T. Maxson, | On Hysteria. |
| Peter B. McKay, | On Diarrhoæ. |
| Edwin G. Meek, | On Syphilis. |
| Gideon C. Paramore, | On Latent Pneumonia. |
| James C. Patterson, | On Tonsillitis. |
| Charles H. Richings, | On Diet. |
| John H. Warren, | On Pathomania. |
| Jerome F. Weeks, | On the Anatomy of the Eye, with some of its Diseases. |

The Honorary Degree of Doctor of Medicine was confered upon Dr. Thomas Hall, of Toulon ,Illinois; anDr. d James H. Buell, of Williamsport, Indiana.

